# Associations of lifestyle characteristics with circulating immune markers in the general population based on NHANES 1999 to 2014

**DOI:** 10.1038/s41598-024-63875-2

**Published:** 2024-06-11

**Authors:** Linfen Guo, Yating Huang, Jing He, Deng Li, Wei Li, Haitao Xiao, Xuewen Xu, Yange Zhang, Ru Wang

**Affiliations:** Department of Plastic and Burns Surgery, West China Hospital, Sichuan University, 37 Guoxuexiang, Chengdu, 610041 China

**Keywords:** Leisure-time physical activity, Diet quality, Alcohol consumption, Cigarettes smoking, Immune-inflammatory markers, NHANES, Immunology, Risk factors

## Abstract

Lifestyles maybe associated with the immune and inflammatory state of human body. We aimed to comprehensively explore the relationship between lifestyles and circulating immune-inflammatory markers in the general population. Data from NHANES 1999–2014 was used. Lifestyle factors included leisure-time physical activity (LTPA), diet quality (Healthy Eating Index-2015, HEI-2015), alcohol consumption, cigarettes smoking, sleep hour and sedentary time. Immune makers included C-reactive protein (CRP), neutrophil–lymphocyte ratio (NLR), systemic immune-inflammation index (SII), platelet–lymphocyte ratio (PLR) and monocyte–lymphocyte ratio (MLR). Generalized linear regression models were used to adjust confounders. Regressions of restricted cubic splines were utilized to evaluate the potentially non-linear relationships between exposures and outcomes. As results, HEI was negatively associated with CRP (P < 0.001), SII (P < 0.001), and NLR (P < 0.001). Cigarettes per day was positively associated with CRP (P < 0.001), SII (P < 0.001), and NLR (P = 0.008). Alcohol consumption was negatively associated with CRP (P < 0.001), but positively associated with PLR (P = 0.012) and MLR (P < 0.001). Physical activity was negatively associated with CRP (P < 0.001), SII (P = 0.005), and NLR (P = 0.002), but positively associated with PLR (P = 0.010). Participants with higher healthy lifestyle score had significantly lower CRP, SII and NLR (all P values < 0.05). Most of the sensitivity analyses found similar results. In conclusion, we found significant associations between lifestyles and immune markers in the general population, which may reflect a systemic inflammatory response to unhealthy lifestyles.

## Introduction

Hematological indicators such as lymphocytes, neutrophils, monocytes, and platelets count can reflect the inflammatory condition of the human body. Different combinations of these markers yielded various inflammatory markers including neutrophil–lymphocyte ratio (NLR), platelet–lymphocyte ratio (PLR), systemic immune-inflammation index (SII, calculated according to neutrophil, lymphocyte, and platelet counts), and monocyte-lymphocyte ratio (MLR)^1,2^. Circulating immune-inflammatory markers such as C-reactive protein (CRP), NLR, MLR, PLR and SII were widely-used to evaluate the immune system response^2–7^. Participants with different health status or health conditions typically exhibited different values in these immune or inflammatory indexes, which were associated with disease prognoses and treatment plans^8–12^. For example, MLR had a good predictive value for cardiovascular mortality in ambulatory adults without already-existed cardiovascular disease^1^. NLR could independently predict cardiovascular risk and all-cause mortality^13^.

Multiple lifestyle factors such as diet, physical activity, alcohol consumption and tobacco smoking were associated with the risk of multiple human diseases in published literatures^14–19^. Existed evidence also indicated that healthy lifestyle had an impact on serum C-reactive protein concentrations in diverse types of diseases^20,21^. Moderate physical activity likely reduced pro-inflammatory monocytes in patients with atherosclerosis, which were related to plaque vulnerability^22^. Cigarette smoke exposure consistently showed significant exposure–response and dose–response relationships with white blood cell count in both males and females^23^. However, previous studies have not clearly and comprehensively illustrated the effect of lifestyles on human immune-inflammatory status in studies with large sample sizes. In this study, we utilized general population data from the 1999–2014 National Health and Nutrition Examination Survey (NHANES) to examine the associations of lifestyle factors with immune-inflammatory markers, aiming to reveal the potential impact of lifestyles on immune system responses.

## Methods

### Study population

**Figure 1:**
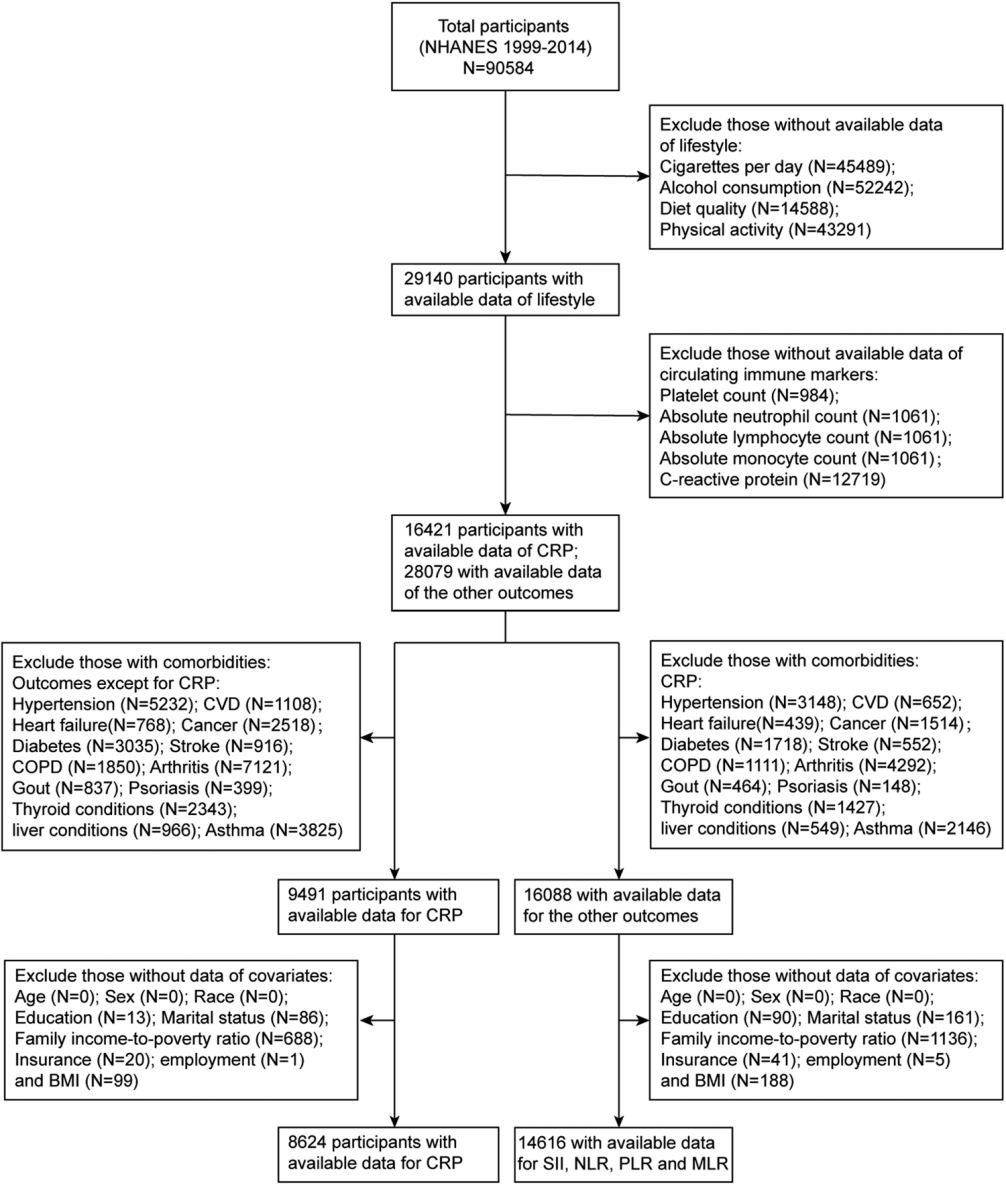
Flow chart of participants who met the inclusion criteria and were included in this study.

The NHANES is conducted by the National Center for Health Statistics (NCHS) of the Centers for Disease Control and Prevention (CDC), and it is a multistage, large-scale, continuous and nationally representative health survey of the noninstitutionalized US civilians. All of the survey protocols were approved by the research ethics review board at the NCHS, and written informed consents were obtained from all the participants. The survey was conducted in accordance with the ethical standards of the 1964 declaration of Helsinki and its later amendments. In this study, we used data from NHANES 1999–2014 data set. Considering that data on variables such as alcohol consumption and smoking was exclusively collected and released for participants aged 20 years or older in partial years, we only included participants aged > 20 years at the baseline survey. This study excluded those with diseases (including hypertension, cardiovascular diseases, heart failure, cancer, diabetes, stroke, asthma, chronic obstructive pulmonary disease, arthritis, gout, psoriasis, thyroid conditions and liver conditions), as these diseases were associated with the exposures and outcomes of the current study, thus, diseases might be a significant confounder or interactive factor for the associations. Then participants with missing data of exposures (lifestyle factors) and outcomes (circulating immune-inflammatory markers) were excluded. Participants without available data of co-variables needed in the multivariable-adjusted models were also excluded. All NHANES 1999–2014 participants aged > 20 years from whom the previously described data could be extracted were deemed to meet the inclusion criteria. Finally, our study included 14,616 participants for the outcome of PLR, SII, NLR and MLR (cohort 1), and 8624 participants for the outcome of CRP (cohort 2). A flow chart of the sample selection process of this study is presented in Fig. 1 in detail.

### Measurement of lifestyle factors

Physical activity was evaluated based on the physical activity questionnaires (PAQs). Leisure-time physical activity (LTPA) was measured, and it was presented as metabolic equivalent time in the analyses^14^. Some different assessment questions were used of year 1999–2006 and 2007–2014, thus, these two periods were evaluated separately. From 1999 to 2006, the frequency of different types of physical activities were provided in the PAQs (https://wwwn.cdc.gov/Nchs/Nhanes/2003-2004/PAQIAF_C.htm# Component_Description). Duration (minutes) of each activity at each time was also provided in the website. Metabolic equivalent (MET) score (intensity) for the activity was also obtained. After 2007, the average time of activity per day and the total days of LTPA per month (moderate or vigorous) were provided at the NHANES website. Four and eight of MET scores were given to moderate and vigorous physical activity, respectively. Finally, the metabolic equivalent times for each participant were calculated by multiplying time (hour) spent on each activity and its metabolic equivalent score from 1999 to 2014.

The diet quality was assessed by Healthy Eating Index-2015 (HEI-2015) score which was recommended by the US Department of Agriculture (USDA) to evaluate the adherence to the dietary guidelines of 2015–2020 Dietary Guidelines for Americans (DGA)^24^. Based on the HEI-2010, HEI-2015 has been developed by replacing empty calories with saturated fat and added sugar, with the result being 13 components (total fruits, whole fruits, total vegetables, greens and beans, dairy foods, whole grains, refined grains, total protein foods, seafood and plant proteins, fatty acids, saturated fats, added sugars, and sodium). The HEI yielded a total score that ranged between 0 and 100 points, and a higher score reflected healthier eating. The total HEI-2015 scores were computed based on the 24-h dietary recall data and the USDA Food Patterns Equivalents Database (MyPyramid Equivalents Database was used for data of year 2001–2004), and the SAS 9.4 software was used to achieve the calculation.

Alcohol consumption was presented as the average number of drinks per day in a period of 12 months, which was calculated based on the reported frequency (days) of alcohol drinking over the past 12 months and average drinks per day on those days that drank alcoholic beverages^25^. One drink referred to a 1.5-oz of liquor, a 5-oz glass of wine, or a 12-oz beer. Cigarettes per day was utilized to represent the intensity of smoking of the participants^26^. For the non-current smokers (ever smokers), cigarettes per day of the smoked days were used. Sleep hour per day (year 2007–2014) was confirmed based on the question: “How much sleep do you usually get at night on weekdays or workdays”. Sedentary hour per day (year 2007–2014) was calculated by summing up time of sitting at school, at home, getting to and from places, traveling in a car or bus, or with friends including time spent sitting at a desk, reading, playing cards, using a computer, or watching television. A healthier lifestyle score was defined as HEI score above the median, physical activity above the median, alcohol use below the median and cigarettes per day below the median. For each healthier lifestyle factor, the participants received 1 point if they met the criterion for the healthy lifestyle score, or 0 points otherwise. The sum of the 4 factors constituted a final healthy lifestyle score (HLS) of 0, 1, 2, 3, or 4, and a higher score indicated a healthier lifestyle. In sensitivity analysis, sleep hour (6–8 h per day was defined as a healthier lifestyle) and sedentary time (below median was defined as a healthier lifestyle) were also included in the HLS, thus, the sum of 6 factors included a HLS of 0–6.

### Definition of immune inflammation biomarkers

Blood sample collection and processing were based on instructions introduced in the NHANES Laboratory/Medical Technologists Procedures Manual (LPM). Latex-enhanced nephelometry was used to quantification of CRP, and details were shown in the NHANES website (https://wwwn.cdc.gov/nchs/data/nhanes/2007-2008/labmethods/crp_e_met.pdf). Hematological parameters, including platelet count (PLT) and differential white cell count (absolute lymphocyte count, absolute neutrophil count, and absolute monocyte count) were evaluated using automated hematology analyzing devices (The Coulter HMX Hematology Analyzer) and were shown as 1000 cells/µL. The SII level was defined as platelet count × neutrophil count/lymphocyte count. We calculated the NLR by dividing the absolute neutrophil count by the absolute lymphocyte count. Similarly, MLR was computed by dividing the absolute monocyte count by the absolute lymphocyte count, and PLR was calculated by PLT/absolute lymphocyte count. Laboratory Procedure Manual of the Complete Blood Count was shown in the NHANES website (https://wwwn.cdc.gov/nchs/data/nhanes/2007-2008/labmethods/cbc_e_met.pdf).

### Covariates

Covariates that may affect the association between lifestyles and immune-inflammatory biomarkers were included in this study, including age (years), sex (female/male), race/ethnicity (Mexican American/other Hispanic/non-Hispanic White/non-Hispanic Black/other race, including multi-racial), education level (less than 9th grade/9–12th grade or equivalent/college or above), employment (employed/unemployed), family income-to-poverty ratio, insurance (insured/uninsured) and marital status (married or cohabited/widowed/divorced or separated/unmarried).

### Data analysis

Participants’ characteristics were presented as mean ± standard deviation (SD) for continuous variables or as frequency (%) for categorical or ordinal variables. Median (min–max) was also used if distributions of the continuous variables were non-normal. We compared differences between groups of categorical and continuous variables using the chi-squared and Student’s t-tests (replaced with Kruskal–Wallis test for non-normally distributed data), respectively. Because of the skewed distributions of LTPA, cigarettes per day and average drinks per day, these variables were ln-transformed to approximate a normal distribution when they were used as continuous variables in analyses. According to the NHANES Analytic and Reporting Guidelines, we incorporated the combined sample weights for 1999–2014 in the statistical analyses.

We used generalized linear regression models to explore the relationships between individual lifestyle factors or healthy lifestyle score, and the immune inflammation biomarkers. Models were adjusted for age, sex, race, education, family income-to-poverty ratio, marital status, employment and insurance. Regressions of restricted cubic splines (RCSs) were utilized to evaluate the potentially non-linear relationships between exposures and outcomes. The Akaike information criterion (AIC) criterion was utilized to confirm the most suitable knots corresponding to the smallest AIC. P-non-linear values were produced using a the ‘anova’ function in the ‘rms’ package to explore the statistical significance of dose–response associations. We conducted subgroup analyses by sex (male/female), age (20–39 years/40–59 years/≥ 60 years), race (Mexican American/other Hispanic/non-Hispanic White/non-Hispanic Black/other race), and year of NHANES data (1999–2006/2007–2014). Three sensitive analyses were performed. First, associations between lifestyle factors and immune-inflammatory markers were analyzed in multivariate regressions after excluding those without any LTPA, alcohol consumption or cigarette use; Second, survey-weighted regressions were carried out only using data from NHANES 2007–2014. For data of 2007–2014, except for the above four factors, sleep hour per day and sedentary time (hour per day) were also included as lifestyle factors; Third, based on data of NHANES 2007–2014, regressions were performed after excluding those without any LTPA, alcohol consumption or cigarette use. R software 4.4.1 was used for analyses and P values less than 0.05 were considered statistically significant.

### Ethics approval and consent to participate

All of the protocols (Protocol #98-12, Protocol #2005-06, Continuation of Protocol #2005-06, Protocol #2011-17 and Continuation of Protocol #2011-17) of the survey were approved by the research ethics review board at the National Center for Health Statistics, and written informed consents were obtained from all the participants.

## Results

### Basic characteristics of study participants

The baseline demographic information for all participants was shown in Table 1. Among 14,616 participants in cohort 1 (outcomes included SII, NLR, PLR and MLR), 52.1% were female, 55.0% were non-Hispanic White, and mean age was 55.7 years (SD, 16.9). 52.1% participants had an education level of college or above, and 84.5% were insured. Additionally, 60.6% participants were married or cohabiting. Values of lifestyle indicators (HEI, cigarettes per day, average drinks per day and metabolic equivalent times of LTPA) and circulating immune markers were presented in Table 1 detailed. Among 8624 participants in cohort 2, the basic characteristics were similar to cohort 1. Of these participants, 51.8% were female, 57.5% were non-Hispanic White, and the mean age was 56.0 years (SD, 17.0). In addition, 49.2% had an education level of college or above, 84.5% were insured, and 61.5% were married or cohabiting.

**Table 1:** Basic demographics of the study sample.

Variables	Cohort 1 (N = 14,616)	Cohort 2 (N = 8624)
Demographic parameters		
Age, years	55.7 ± 16.9	56.0 ± 17.0
Sex		
Male	7005 (47.9%)	4153 (48.2%)
Female	7611 (52.1%)	4471 (51.8%)
Race/ethnicity, %		
Mexican American	1789 (12.2%)	1163 (13.5%)
Other Hispanic	1015 (6.9%)	562 (6.5%)
Non-Hispanic White	8036 (55.0%)	4960 (57.5%)
Non-Hispanic Black	2924 (20.0%)	1621 (18.8%)
Other race—including multi-racial	852 (5.8%)	318 (3.7%)
Socioeconomic status		
Education levels, %		
Less than 9th grade	1461 (10.0%)	974 (11.3%)
9–12th grade or equivalent	5547 (38.0%)	3411 (39.6%)
College or above	7608 (52.1%)	4239 (49.2%)
Employment		
Employed	6884 (47.1%)	4053 (47.0%)
Unemployed	7732 (52.9%)	4571 (53.0%)
Family income-to-poverty ratio	2.6 ± 1.6	2.7 ± 1.6
Insurance		
Insured	12,353 (84.5%)	7284 (84.5%)
Uninsured	2263 (15.5%)	1340 (15.5%)
Marital status		
Married/cohabited	8850 (60.6%)	5308 (61.5%)
Widowed	1631 (11.2%)	997 (11.6%)
Divorced/separated	2297 (15.7%)	1325 (15.4%)
Unmarried	1838 (12.6%)	994 (11.5%)
Anthropometry		
BMI, kg/m^2^	29.8 ± 7.0	29.6 ± 6.8
Lifestyle indicators		
HEI score	53.7 ± 13.8	53.5 ± 13.8
Cigarettes per day	1.0 (0.0–95.0)	1.0 (0.0–95.0)
Alcohol consumption*	0.0 (0.0–28.3)	0.0 (0.0–28.3)
Physical activity**	7.0 (0.0–563.1)	7.0 (0.0–563.1)
Circulating immune markers		
C-reactive protein (mg/dL)		0.2 (0.0–25.4)
Total leukocyte count (1000 cell/μL)	7.3 ± 2.6	
Absolute lymphocyte count (1000 cell/μL)	2.1 ± 1.5	
Absolute neutrophil count (1000 cell/μL)	4.3 ± 1.8	
Absolute monocyte count (1000 cell/μL)	0.6 ± 0.2	
Neutrophil–lymphocyte ratio	2.3 ± 1.3	
Systemic immune-inflammation index	569.3 ± 369.1	
Monocyte–lymphocyte ratio	0.3 ± 0.1	
Platelet–lymphocyte ratio	131.6 ± 55.2	

*HEI* healthy eating index, *BMI* body mass index.

*Average drinks per day.

**Metabolic equivalent times, calculated by multiplying time spent on each activity (hour) and its metabolic equivalent score.

### Associations between individual lifestyle factors and circulating immune markers

The results from the linear regression analysis regarding the associations of HEI, smoking (cigarettes per day), alcohol consumption (average drinks per day) and physical activity (metabolic equivalent times) with immune biomarkers were reported in Table 2. After adjusting for confounders, we found that HEI score was significantly and negatively associated with CRP (P < 0.001), SII (P < 0.001), and NLR (P < 0.001). Cigarettes per day was positively associated with CRP (P < 0.001), SII (P < 0.001), and NLR (P = 0.008). Alcohol consumption was negatively associated with CRP (P < 0.001), but positively associated with PLR (P = 0.012) and MLR (P < 0.001). Physical activity (LTPA) was negatively associated with CRP (P < 0.001), SII (P = 0.005), and NLR (P = 0.002), but positively associated with PLR (P = 0.010). Regressions were also performed after dividing the exposures variables into two categories with the median values, then we found that some associations became insignificant (such as associations between alcohol drinking and PLR, or MLR), which may be caused by the potentially non-linear relationships between them (Table 2).

**Table 2:** Associations between lifestyle factors and immune-inflammatory markers based on survey-weighted regression.

Outcomes	Adjusted β (95% CI)	P value	Adjusted β (95% CI)	P value	Adjusted β (95% CI)	P value	Adjusted β (95% CI)	P value
	HEI score		Cigarettes per day		Average drinks per day		Physical activity**	
C-reactive protein (mg/dL)	− **0.0114 (**− **0.0139,** − **0.0089)**	** < 0.001**	**0.0074 (0.0050, 0.0098)**	** < 0.001**	− **0.0478 (**− **0.0711,** − **0.0244)**	** < 0.001**	− **0.0030 (**− **0.0039,** − **0.0020)**	** < 0.001**
Platelet-lymphocyte ratio	− 0.0002 (− 0.0007, 0.0004)	0.545	− 0.0005 (− 0.0012, 0.0001)	0.125	**0.0101 (0.0023, 0.0178)**	**0.012**	**0.0003 (0.0001, 0.0005)**	**0.010**
Systemic immune-inflammation index	− **0.0024 (**− **0.0032,** − **0.0017)**	** < 0.001**	**0.0015 (0.0007, 0.0023)**	** < 0.001**	0.0004 (− 0.0104, 0.0113)	0.937	− **0.0005 (**− **0.0008,** − **0.0002)**	**0.005**
Neutrophil–lymphocyte ratio	− **0.0012 (**− **0.0018,** − **0.0005)**	** < 0.001**	**0.0009 (0.0002, 0.0016)**	**0.008**	0.0047 (− 0.0051, 0.0144)	0.350	− **0.0005 (**− **0.0009,** − **0.0002)**	**0.002**
Monocyte–lymphocyte ratio	0.0003 (− 0.0003, 0.0009)	0.312	− 0.0002 (− 0.0008, 0.0003)	0.405	**0.0218 (0.0150, 0.0285)**	** < 0.001**	− 0.0001 (− 0.0003, 0.0001)	0.335

	HEI-high vs. HEI-low		Ever-smoking vs. never-smoking		Ever-drinking vs. never-drinking		PA-high vs. PA-low	
C-reactive protein (mg/dL)	− **0.2682 (**− **0.3371,** − **0.1992)**	** < 0.001**	0.0200 (− 0.0711, 0.1110)	0.669	− **0.2021 (**− **0.2681,** − **0.1360)**	** < 0.001**	− **0.2386 (**− **0.3119,** − **0.1653)**	** < 0.001**
Platelet–lymphocyte ratio	0.0038 (− 0.0105, 0.0182)	0.601	− **0.0450 (**− **0.0622,** − **0.0279)**	** < 0.001**	0.0068 (− 0.0132, 0.0267)	0.506	0.0077 (− 0.0102, 0.0256)	0.400
Systemic immune-inflammation index	− **0.0420 (**− **0.0611,** − **0.0229)**	** < 0.001**	**0.0238 (0.0027, 0.0449)**	**0.030**	− 0.0210 (− 0.0466, 0.0046)	0.110	− **0.0479 (**− **0.0710,** − **0.0249)**	** < 0.001**
Neutrophil–lymphocyte ratio	− **0.0185 (**− **0.0344,** − **0.0026)**	**0.025**	0.0141 (− 0.0036, 0.0318)	0.117	− 0.0081 (− 0.0299, 0.0137)	0.468	− **0.0552 (**− **0.0752,** − **0.0353)**	** < 0.001**
Monocyte–lymphocyte ratio	0.0141 (− 0.0008, 0.0289)	0.066	− **0.0213 (**− **0.0359,** − **0.0068)**	**0.005**	0.0077 (− 0.0067, 0.0220)	0.299	− **0.0213 (**− **0.0372,** − **0.0055)**	**0.010**

*HEI* healthy eating index, *PA* physical activity.

**Metabolic equivalent times, calculated by multiplying time spent on each activity (hour) and its metabolic equivalent score. These variables were adjusted: age, sex, race, education, family income-to-poverty ratio, marital status, employment and insurance. The other three lifestyle factors except for the analyzed one were also adjusted. All lifestyle factors were ln-transformed except for HEI. All outcomes were ln-transformed.

Significant values are in bold.

**Figure 2:**
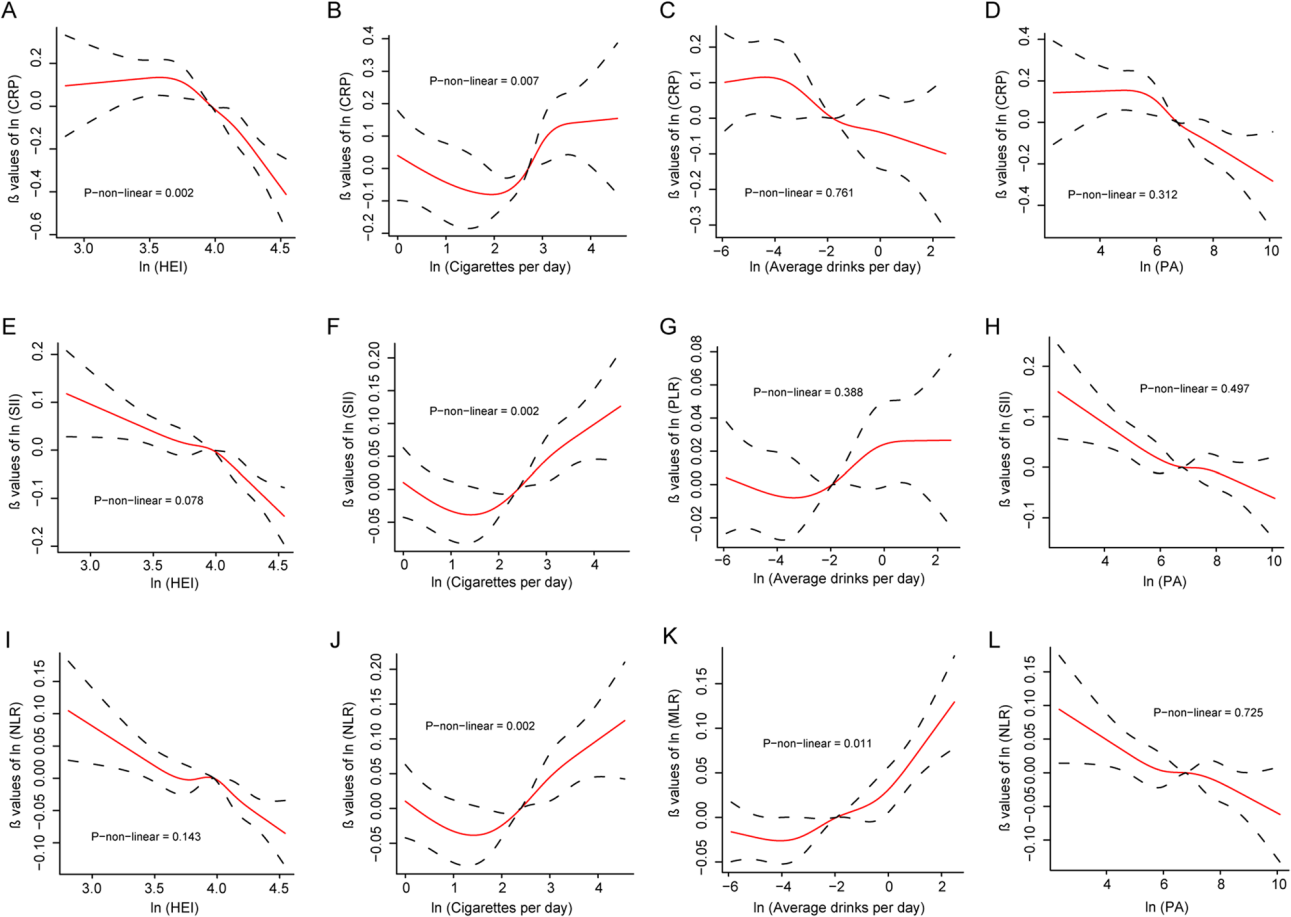
Curves of restricted cubic splines between lifestyles and outcomes. (**A–D**), associations between HEI (**A**), cigarettes per day (**B**), average drinks per day (**C**), and physical activity (PA) (**D**) and CRP; (**E–H**), associations between HEI (**E**), cigarettes per day (**F**), average drinks per day (**G**), and PA (**H**) and SII; (**I–L**), associations between HEI (**I**), cigarettes per day (**J**), average drinks per day (**K**), and PA (**L**) and NLR.

As depicted in Fig. 2, among statistically significant associations found above, we analyzed dose–response relationships between lifestyle factors and outcomes. We observed linear relationships between HEI and SII, and HEI and NLR (both P-non-linear values > 0.05). In contrast, HEI was significantly and negatively associated with CRP only in participants with higher HEI score (P-non-linear = 0.002) (Fig. 2A). Non-linear J-shaped relationships were observed between cigarettes per day and CRP, SII and NLR (all values of P-non-linear < 0.05) (Fig. 2B,F,J). We found that the positive associations between smoking and outcomes were mainly significant in those with higher number of cigarettes use. Statistically significant associations for alcohol consumption and outcomes were linear except for average drinks per day and MLR (P-non-linear = 0.011). Finally, all negative associations were linear between physical activity and CRP, SII and NLR (all values of P-non-linear > 0.05) (Fig. 2D,H,L). Except for the statistically significant associations in Fig. 2, the other RCS curves of the associations between lifestyles and outcomes were shown in Fig. S1.

### Associations between healthy lifestyle score and immune-inflammatory markers

Next, we explored the overall effect of lifestyle (HLS) on outcomes. As shown in Table 3, participants with higher HLS had significantly lower CRP level, lower SII and lower NLR. For example, compared to HLS 0–1, cases with HLS 3–4 had lower CRP, SII and NLR (all P values < 0.05). However, cases with HLS 3–4 had higher PLR compared to HLS 0–1 (P = 0.002), which may be associated with the non-linear U-shaped relations between cigarettes per day and PLR, and between LTPA and PLR (both P-non-linear values < 0.05) (Fig. S1B,D). Besides, increased LTPA was associated with a higher PLR, which was different from the other trends of LTPA-related associations. In contrast, no significant associations were observed between HLS and MLR.

**Table 3:** Associations between healthy lifestyle score and immune-inflammatory markers based on survey-weighted regression.

Outcomes	HLS 2 vs. HLS 0–1		HLS 3–4 vs. HLS 0–1	
	Adjusted β (95% CI)	P value	Adjusted β (95% CI)	P value
C-reactive protein (mg/dL)	− **0.1195 (**− **0.1977,** − **0.0413)**	**0.004**	− **0.3213 (**− **0.4085,** − **0.2342)**	** < 0.001**
Platelet–lymphocyte ratio	0.0082 (− 0.0107, 0.0271)	0.397	**0.0331 (0.0127, 0.0536)**	**0.002**
Systemic immune-inflammation index	− **0.0418 (**− **0.0936,** − **0.0206)**	** < 0.001**	− **0.0690 (**− **0.0936,** − **0.0444)**	** < 0.001**
Neutrophil–lymphocyte ratio	− **0.0365 (**− **0.0540,** − **0.0189)**	** < 0.001**	− **0.0551 (**− **0.0752,** − **0.0349)**	** < 0.001**
Monocyte–lymphocyte ratio	− 0.0114 (− 0.0296, 0.0068)	0.221	− 0.0025 (− 0.0234, 0.0185)	0.816

These variables were adjusted: age, sex, race, education, family income-to-poverty ratio, marital status, employment and insurance.

*HLS* healthy lifestyle score.

Significant values are in bold.

Subgroup analyses for the associations of HLS with immune markers stratified by various factors were presented in Tables S1 and S2. No significant interactions were observed for the HLS in relation to immune-inflammatory markers when the participants were stratified by age, sex and race (Table S1). Though statistical significances were not obvious in some specific subgroups, it may be caused by the decreased sample size in these subgroups after stratification. Given the potential differences of data collection, we performed subgroup analyses based on data cycles (year 1999–2006 and year 2007–2014). The results also did not show significant interaction of data cycles on the associations between HLS and outcomes (Table S2).

### Sensitivity analysis

In the first sensitivity analysis, we performed analyses after excluding participants without any LTPA, alcohol consumption or cigarette use. Finally, only participants with both LTPA, smoking and alcohol drinking were analyzed in multivariate regressions. In this subpopulation, we found that results were basically consistent with the primary regressions (Table S3). HEI score was found to be significantly and negatively associated with CRP (P < 0.001), SII (P < 0.001), and NLR (P = 0.029). Similarly, alcohol drinking was positively correlated with PLR (P = 0.021) and MLR (< 0.001). In addition, the beneficial role of LTPA and the harmful effect of cigarettes use were still shown in the sensitivity analysis, but the statistical significance was not observed (Table S3). In the second sensitivity analysis, we focused on participants in NHANES 2007–2014, and six types of lifestyle factors were enrolled including LTPA, HEI, cigarettes per day, average drinks per day, sleep hour and sedentary time per day. The effect of LTPA, HEI, cigarettes per day and alcohol drinking were similar to those reported above. For example, alcohol drinking was associated with the decrease of CRP level. Notably, for sleep hour, no significant associations were found when it was treated as a continuous variable. However, after we divided participants into two groups (6–8 h vs. < 6 h or > 8 h), participants who slept 6–8 h per day were found to be associated with lower CRP levels (P = 0.016) (Table S4). For sedentary time, we found that it was significantly associated with higher SII, NLR and MLR (all P values < 0.05). However, it was negatively associated with PLR in analysis using sedentary time as a continuous variable (P = 0.005). For data of 2007–2014, the total HLS ranged from 0 to 6. Compared to HLS 0–1, participants with higher HLS 5–6 or HLS 3–4 had lower CRP, SII and NLR levels (Table 4). In the third sensitivity analysis, after excluding those without LTPA, smoking and alcohol use in NHANES 2007–2014, we found that the HEI was still associated with the CRP, SII and NLR levels (all P values < 0.05). Alcohol use was still associated with PLR (P = 0.009) and MLR (< 0.001) (Table S5).

**Table 4:** Associations between number of healthy lifestyle characteristics and immune-inflammatory markers based on survey-weighted regression in NHANES 2007–2014.

Outcomes	HLS 2 vs. HLS 0–1		HLS 3–4 vs. HLS 0–1		HLS 5–6 vs. HLS 0–1	
	Adjusted β (95% CI)	P value	Adjusted β (95% CI)	P value	Adjusted β (95% CI)	P value
C-reactive protein (mg/dL)	− 0.1227 (− 0.2781, 0.0326)	0.133	− **0.2807 (**− **0.4158,** − **0.1457)**	** < 0.001**	− **0.5068 (**− **0.6556,** − **0.3580)**	** < 0.001**
Platelet-lymphocyte ratio	− 0.0017 (− 0.0309, 0.9095)	0.910	0.0242 (− 0.0025, 0.0785)	0.078	0.0279 (− 0.0099, 0.1499)	0.150
Systemic immune-inflammation index	− 0.0251 (− 0.0729, 0.3095)	0.309	− **0.0595 (**− **0.0973, 0.0035)**	**0.004**	− **0.1190 (**− **0.1759, 0.0002)**	** < 0.001**
Neutrophil–lymphocyte ratio	− 0.0277 (− 0.0712, 0.2199)	0.220	− **0.0504 (**− **0.0833, 0.0044)**	**0.004**	− **0.1093 (**− **0.1564, 0.00004)**	** < 0.001**
Monocyte–lymphocyte ratio	− 0.01300 (− 0.0500, 0.4956)	0.496	− 0.0102 (− 0.0388, 0.4874)	0.487	− 0.0321 (− 0.0751, 0.1506)	0.151

These variables were adjusted: age, sex, race, education, family income-to-poverty ratio, marital status, employment and insurance.

*HLS* healthy lifestyle score.

Significant values are in bold.

## Discussion

In this study, we performed a comprehensive analysis to explore the associations between lifestyles and circulating immune-inflammatory markers. Our results firstly indicated that different types of lifestyle factors were associated with distinct immune-inflammatory markers. HEI (negatively), smoking (positively) and LTPA (negatively) were associated with CRP, SII and NLR levels, whereas alcohol drinking was positively associated with PLR and MLR. Second, linear relationships were found for most of the associations, and the non-linear associations were mainly found between cigarettes use and outcomes. Third, higher healthy lifestyle score was associated with lower CRP, SII and NLR levels in both of the total participants (four-factor HLS), and participants from NHANES 2007–2014 (six-factor HLS). Fourth, some other associations were observed. For example, alcohol drinking was associated with lower CRP level (Table 2); LTPA was positively associated with PLR (Table 2); Sleep of 6–8 h was associated with lower CRP level compared to < 6 h or > 8 h (Table S4). Considering that lifestyle risk factors were potentially modifiable, these findings may have significant implications for approaches to maintain the corresponding immune-inflammatory markers at optimal levels.

Immune-inflammatory markers were previously studied and widely used in predicting treatment outcomes or long-term prognosis of various diseases. For example, SII was a prognostic combination marker in cases with a variety of cancers, such as gastrointestinal cancer, lung cancer and germ-cell cancer^27–30^. Besides, NLR, PLR and MLR have been used as inflammatory markers to predict outcomes and response to treatment in various malignancies. For example, NLR and PLR was associated with the short-term, recurrence-free an overall survival of colorectal cancer^31^. Additionally, CRP was another serum inflammatory marker that has been studied in a number of infections and also used as a biomarker in cancer^32,33^. In addition to cancer, these markers were also associated with the development and prognosis of a series of non-neoplastic diseases, such as cardiovascular diseases, bacterial or viral infectious diseases (e.g., COVID-19) and autoimmune diseases (e.g., rheumatoid arthritis)^34–36^. Our results indicated that lifestyles should also be considered when using the prognostic or predictive function of immune-inflammatory markers, given that lifestyles may be also associated with the levels of these markers.

Some of our results were consistent with several previous studies. Howard et al. observed significant associations between lifestyle factors and NLR in the general population^37^. However, they did not include some important lifestyle factors such as HEI, and the dose-dependent relationships between lifestyle factors and NLR was not analyzed. Moreno-Franco et al. demonstrated that cooking and food preservation patterns, which impact diet quality and drinking behavior, had a relationship with inflammatory biomarkers^38^. Numerous studies have also shown that adherence to a specific dietary pattern, such as the Mediterranean dietary pattern (characterized by a high consumption of fresh and not processed foods), was associated with metabolic and inflammatory biomarkers^39–44^. Based on these studies, we furtherly found that HEI-2015 was significantly associated with CRP, SII and NLR in the general population of US. The previous studies found that moderate-to-vigorous physical activity, but not sedentary behavior, were independently related to reduced odds of elevated CRP^45,46^, which was consistent with results in the current study. In addition, more time spent in recreational physical activity was found to be negatively associated with the SII in the NHANES cohort of 26,254 participants^47^. In a randomized controlled trial, patients with childhood cancer undergoing treatment who received 6–8 weeks of supervised exercise intervention had significantly lower SII compared to controls^48^. Similarly, a 3-week high-intensity interval training intervention in patients with multiple sclerosis resulted in lower SII and NLR^49^. Additionally, overweight Chinese male adolescents showed a significant reduction in NLR following a 4-week diet and physical exercise intervention^50^. These studies collectively support the linear negative correlation of LTPA with NLR and SII observed in this study. However, epidemiological studies related to associations between LTPA and PLR were limited. The dose-dependent relationship between LTPA and inflammation indicated that, in the general population, reasonable increase of LTPA could help to attenuate the inflammatory state of the body. It is recommended to increase the frequency and duration of physical activity as physical fitness allows and to choose higher intensity sports such as basketball, hiking, and field hockey. The anti-inflammatory effects of LTPA should be evaluated furtherly to understand the effect of different exercise modalities or durations (such as acute or chronic exercise) on human inflammatory status. Notably, the positive associations between LTPA and PLR (Table 2, Table S3) indicated the inflammatory response was also existed after LTPA. A possible explanation for it might be the exercise-dependent mobilization of platelets into the peripheral circulation^51^.

For tobacco smoking, published studies have illustrated its proinflammatory effect using the indicator of CRP. Besides, elevated NLR caused by smoking were observed in previous studies^52,53^. The trends of PLR in smokers were contradictory among those studies^52,53^. In this study, we furtherly used a continuous variable (cigarettes per day) to represent the exposure to smoking, and the J-shaped relationships were observed between smoking and outcomes (significant for CRP, SII and NLR), whereas no significant association was found between smoking and PLR. Future studies should be performed to explain the mechanisms of the non-linear relations between them.

Thrombocytosis and lymphopenia have been associated with systemic inflammatory response and PLR was used as a new marker to combine both hematological markers. Interestingly, alcohol drinking was significantly and positively associated with PLR and MLR in our results, but negatively associated with CRP in our study. Our results indicated that the immune status of the human body after alcohol drinking should be evaluated comprehensively based on all available immune markers. For example, the CRP, PLR or MLR showed different responses after alcohol drinking. However, the detailed algorithms to integrate these markers should be developed in the future. The previously published study showed a U-shaped relation between alcohol consumption and CRP^54^. Our study mainly focused on the healthier population (cases with morbidities were excluded), thus, we could not observe the part of positive association of the U-shape curve. Additionally, based on the previous study, we supposed that both of the proinflammatory and anti-inflammatory effect of alcohol drinking might be associated with the different types or volumes of alcohol beverages. For example, resveratrol in red wine and hops in beer could contribute to an anti-inflammatory effect through inhibition of transcription factors^55–57^. In addition, alcohol related inflammation was also clearly observed, alcohol inflammation inducers mainly derived from alcohol damaged cells and gut microflora, such as lipopolysaccharide^58^. Alcohol metabolism could also activate some inflammation transcription factor, such as nuclear factor-κB^59^. Future studies should explore the anti-inflammatory and proinflammatory mechanisms of different types or volumes of alcohol drinking detailly. Finally, sleep duration between 6–8 h was found to be associated with lower CRP levels, which was consistent with previous study that sleep disturbances are associated with elevated levels of inflammation^60,61^. A possible mechanism underlying this association was the absence of a diurnal rhythm in CRP levels. The hepatic production of CRP was stimulated by cytokines such as IL-6 and IL-17, which are upregulated by sleep deprivation or excessive sleep duration^62–64^.

The strengths of this study are the wealth of lifestyle and immune-inflammatory variables in a large representative sample of the US population. This study is the first to comprehensively examine the relationship between different lifestyle factors and multiple immune inflammation biomarkers (CRP, NLR, PLR, MLR and SII) in a large number of US subjects without diseases. The increase of specific immune or inflammation biomarkers after exposure to different types of lifestyle factors indicated that the pathways related to activation of the immune system or inflammatory response by these lifestyle exposures maybe different. Moreover, the significances of these immune biomarkers were distinct for prediction of disease progression or prognosis, thus, lifestyles linked to the marker should also be considered to be confounding factors when using these biomarkers for prediction. The first limitation of this study was that the cross-sectional design did not allow us to explore the causal relationship between lifestyles and outcomes. Second, mechanisms behind the significant associations found in this study were not studied, and further researches were needed to illustrate them. Third, the nonlinear relations between some associations could not be explained sufficiently. Subsequent studies could conduct RCS analyses at the 5th, 27.5th, 50th, 72.5th, and 95th percentiles of lifestyle variables, with varying degrees of adjustment for covariates^65,66^. Fitted smoothing curves and threshold effect analysis could also be utilized to describe the nonlinear relationships^67^. Fourth, though we constructed the HLS for overall lifestyle evaluation, in the real world, the joint effect of lifestyle was difficult to assess, and future studies should be performed to investigate the overall impact of lifestyle on immune or inflammatory state of the general population. For example, a comprehensive collection of variables reflecting the healthiness of lifestyle in different databases or large population-based cohorts could be analyzed, such as maintaining an appropriate body weight, adequate water intake, regular medical checkups, good hygiene habits, positive psychological state, and so on^68^. Last, although we excluded participants with diseases such as hypertension and cardiovascular diseases, the influence of diseases on immunological parameters cannot be entirely ruled out, as the NHANES database lacks comprehensive information on immunological diseases such as celiac diseases, scleroderma and vitiligo.

To conclude, given the significant associations between lifestyles and immune-inflammatory markers in the general population, modification of lifestyle exposures including alcohol use, physical activity, diet quality, sedentary behavior, short/long sleep duration, and cigarettes smoking for at-risk populations maybe useful to improve the immune or inflammatory state of human body. Besides, the already established association between immune biomarkers and multiple medical conditions indicated that, reducing inflammation by lifestyle improvement may offer a disease-prevention strategy. Finally, the present observations may also provide guidance for future interventional study design by suggesting optimal characteristics for participant matching.

### Supplementary Information

Supplementary Information.

## Data Availability

The National Health and Nutrition Examination Survey (NHANES) data are publicly available at https://www.cdc.gov/nchs/nhanes/index.htm.

## Abbreviations

CRP: C-reactive protein
NLR: Neutrophil–lymphocyte ratio
MLR: Monocyte–lymphocyte ratio
PLR: Platelet–lymphocyte ratio
SII: Systemic immune-inflammation index
NHANES: National Health and Nutrition Examination Survey
NCHS: National Center for Health Statistics
CDC: Control and Prevention
PAQ: Physical activity questionnaires
LTPA: Leisure-time physical activity
MET: Metabolic equivalent
HEI-2015: Healthy Eating Index-2015
USDA: US Department of Agriculture
DGA: Dietary Guidelines for Americans
LPM: Laboratory/Medical Technologists Procedures Manual
