# Supplementary material for: Associations of lifestyle characteristics with circulating immune markers in the general population based on NHANES 1999 to 2014
Source: Sci Rep. 2024 Jun 11;14:13444. doi: 10.1038/s41598-024-63875-2 (PMC11166635; doi:10.1038/s41598-024-63875-2)
Supplement: Supplementary file 1 — Supplementary Information. [file 41598_2024_63875_MOESM1_ESM.docx]

**Associations of** **lifestyle characteristics with** **circulating** **immune markers in the general population based on NHANES 1999 to 2014**

Linfen Guo^1^, Yating Huang^1^, Jing He^1^, Deng Li^1^, Wei Li^1^, Haitao Xiao^1^, Xuewen Xu^1^, Yange Zhang^1^, Ru Wang^1^

^1^Department of Plastic and Burns Surgery, West China Hospital, Sichuan University, Chengdu 610041, China;

Corresponding author: Yange Zhang, Ru Wang

Correspondence: Department of Plastic and Burns Surgery, West China Hospital, Sichuan University, 37 Guoxuexiang, Chengdu 610041, China. E-mail address: zhangyangeplastic@126.com.


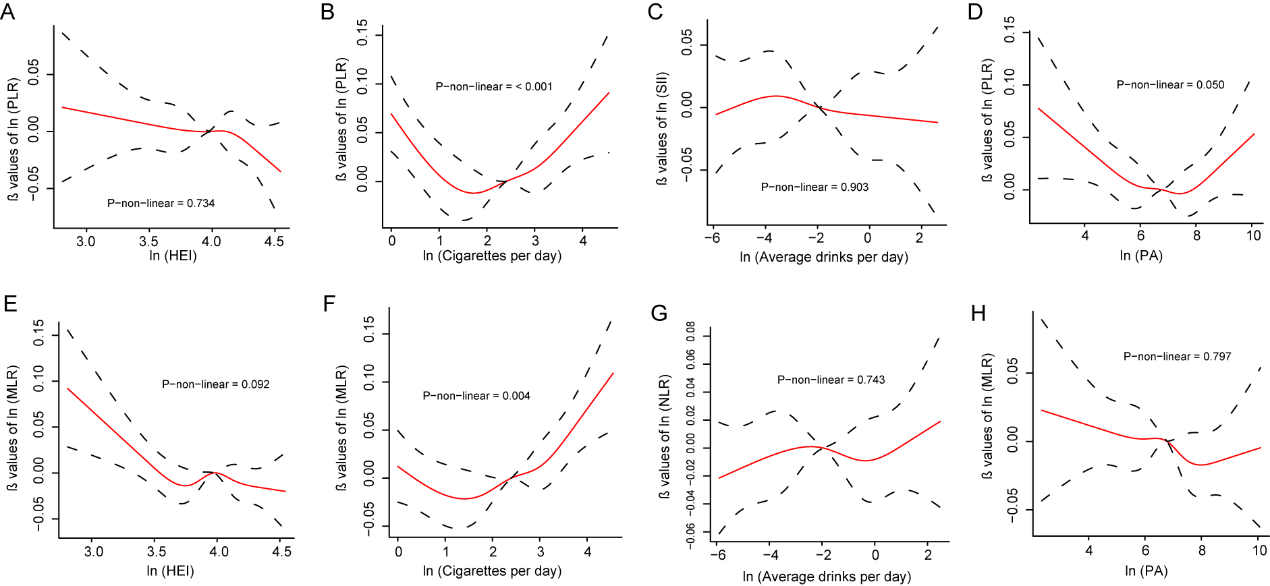


Fig. S1 Curves of restricted cubic splines between lifestyles and outcomes. (A), association between HEI and PLR; (B), association between cigarettes per day and PLR; (C), association between average drinks per day and SII; (D), association between physical activity (PA) and PLR; (E), association between HEI and MLR; (F), association between cigarettes per day and MLR; (G), association between average drinks per day and NLR; (H), association between PA and MLR.

| Table S1 Associations between lifestyle characteristics and immune-inflammation markers stratified by sex, age and race | | | | |  |
| --- | --- | --- | --- | --- | --- |
| Outcomes | HLS 2 vs. HLS 0-1 | | HLS 3-4 vs. HLS 0-1 | |  |
|  | Adjusted β (95%CI) | P value | Adjusted β (95%CI) | P value |  |
| **C-reactive protein (mg/dL)** |  |  |  |  |  |
| Sex |  |  |  |  |  |
| Men | **-0.2090 (-0.2947, -0.1233)** | **<0.001** | **-0.3868 (-0.4821, -0.2916)** | **<0.001** |  |
| Women | -0.0642 (-0.1616, 0.0332) | 0.201 | **-0.2003 (-0.3053, -0.0954)** | **<0.001** |  |
| Age, years |  |  |  |  |  |
| 20-39 y | **-0.2517 (-0.3884, -0.1149)** | **0.001** | **-0.2688 (-0.4106, -0.1270)** | **<0.001** |  |
| 40-59 y | **-0.1303 (-0.2497, -0.0108)** | **0.037** | **-0.3245 (-0.4446, -0.2044)** | **<0.001** |  |
| 60 y | -0.0760 (-0.1790, 0.0270) | 0.153 | **-0.2600 (-0.3765, -0.1434)** | **<0.001** |  |
| Race |  |  |  |  |  |
| Mexican American | 0.1151 (-0.0680, 0.2981) | 0.226 | -0.0005 (-0.2180, 0.2170) | 0.997 |  |
| Other Hispanic | 0.0352 (-0.3048, 0.3751) | 0.841 | 0.0496 (-0.2502, 0.3494) | 0.749 |  |
| Non-Hispanic White | **-0.1655 (-0.2400, -0.0910)** | **<0.001** | **-0.3678 (-0.4497, -0.2860)** | **<0.001** |  |
| Non-Hispanic Black | -0.1207 (-0.2721, 0.0308) | 0.125 | **-0.2052 (-0.3639, -0.0465)** | **0.015** |  |
| Other Race - including multi-racial | -0.1838 (-0.5002, 0.1326) | 0.265 | 0.0744 (-0.2470, 0.3957) | 0.654 |  |
| **Platelet-lymphocyte ratio** |  |  |  |  |  |
| Sex |  |  |  |  |  |
| Men | 0.0113 (-0.0172, 0.0398) | 0.439 | **0.0573 (0.0306, 0.0840)** | **<0.001** |  |
| Women | 0.0067 (-0.0215, 0.0349) | 0.641 | 0.0182 (-0.0108, 0.0472) | 0.221 |  |
| Age, years |  |  |  |  |  |
| 20-39 y | 0.0225 (-0.0119, 0.057) | 0.203 | **0.0516 (0.0201, 0.0832)** | **0.002** |  |
| 40-59 y | 0.0161 (-0.0158, 0.048) | 0.326 | **0.0689 (0.0340, 0.1039)** | **<0.001** |  |
| 60 y | -0.0157 (-0.0452, 0.0139) | 0.301 | -0.0132 (-0.0437, 0.0174) | 0.401 |  |
| Race |  |  |  |  |  |
| Mexican American | -0.0206 (-0.0706, 0.0295) | 0.424 | -0.0042 (-0.0487, 0.0404) | 0.856 |  |
| Other Hispanic | 0.0286 (-0.0406, 0.0977) | 0.422 | 0.0352 (-0.0191, 0.0894) | 0.210 |  |
| Non-Hispanic White | 0.0053 (-0.0169, 0.0274) | 0.644 | **0.0312 (0.0056, 0.0568)** | **0.019** |  |
| Non-Hispanic Black | 0.0158 (-0.029, 0.0607) | 0.491 | **0.0630 (0.0231, 0.1030)** | **0.003** |  |
| Other Race - including multi-racial | 0.0191 (-0.0700, 0.1082) | 0.676 | 0.0334 (-0.0475, 0.1143) | 0.422 |  |
| **Systemic immune-inflammation index** |  |  |  |  |  |
| Sex |  |  |  |  |  |
| Men | **-0.0353 (-0.0679, -0.0028)** | **0.035** | -0.0185 (-0.0556, 0.0186) | 0.332 |  |
| Women | **-0.0405 (-0.0723, -0.0087)** | **0.014** | **-0.0949 (-0.1277, -0.0621)** | **<0.001** |  |
| Age, years |  |  |  |  |  |
| 20-39 y | **-0.0549 (-0.0951, -0.0146)** | **0.009** | **-0.0800 (-0.1293, -0.0306)** | **0.002** |  |
| 40-59 y | -0.0213 (-0.0616, 0.0191) | 0.304 | -0.0292 (-0.0675, 0.0092) | 0.139 |  |
| 60 y | **-0.0523 (-0.0933, -0.0113)** | **0.014** | **-0.0895 (-0.1340, -0.0451)** | **<0.001** |  |
| Race |  |  |  |  |  |
| Mexican American | -0.0736 (-0.1478, 0.0006) | 0.052 | -0.0733 (-0.1469, 0.0004) | 0.051 |  |
| Other Hispanic | -0.0632 (-0.1597, 0.0332) | 0.205 | **-0.1133 (-0.2054, -0.0212)** | **0.020** |  |
| Non-Hispanic White | **-0.0469 (-0.0718, -0.0219)** | **<0.001** | **-0.0741 (-0.1041, -0.0441)** | **<0.001** |  |
| Non-Hispanic Black | -0.0032 (-0.0575, 0.0511) | 0.906 | -0.0345 (-0.0858, 0.0168) | 0.184 |  |
| Other Race - including multi-racial | 0.0021 (-0.1261, 0.1302) | 0.975 | -0.0066 (-0.1160, 0.1028) | 0.906 |  |
| **Neutrophil-lymphocyte ratio** |  |  |  |  |  |
| Sex |  |  |  |  |  |
| Men | **-0.0280 (-0.0548, -0.0011)** | **0.044** | -0.0097 (-0.0372, 0.0179) | 0.492 |  |
| Women | **-0.0354 (-0.0627, -0.0081)** | **0.012** | **-0.0752 (-0.1036, -0.0467)** | **<0.001** |  |
| Age, years |  |  |  |  |  |
| 20-39 y | **-0.0463 (-0.0815, -0.0112)** | **0.011** | **-0.0681 (-0.1122, -0.0241)** | **0.003** |  |
| 40-59 y | -0.0233 (-0.0566, 0.0099) | 0.172 | -0.0236 (-0.0588, 0.0117) | 0.193 |  |
| 60 y | -0.0304 (-0.0621, 0.0013) | 0.063 | **-0.0544 (-0.0894, -0.0193)** | **0.003** |  |
| Race |  |  |  |  |  |
| Mexican American | **-0.0652 (-0.1196, -0.0108)** | **0.022** | **-0.0816 (-0.1384, -0.0249)** | **0.007** |  |
| Other Hispanic | **-0.0870 (-0.1707, -0.0034)** | **0.042** | **-0.1337 (-0.2241, -0.0432)** | **0.005** |  |
| Non-Hispanic White | **-0.0387 (-0.0588, -0.0185)** | **<0.001** | **-0.0529 (-0.0753, -0.0306)** | **<0.001** |  |
| Non-Hispanic Black | 0.0059 (-0.0414, 0.0532) | 0.808 | -0.0426 (-0.0888, 0.0037) | 0.075 |  |
| Other Race - including multi-racial | -0.0338 (-0.1391, 0.0714) | 0.531 | -0.0160 (-0.1066, 0.0745) | 0.730 |  |
| **Monocyte-lymphocyte ratio** |  |  |  |  |  |
| Sex |  |  |  |  |  |
| Men | -0.0102 (-0.0368, 0.0165) | 0.456 | 0.0091 (-0.0156, 0.0338) | 0.471 |  |
| Women | -0.0083 (-0.0340, 0.0175) | 0.531 | -0.0050 (-0.0371, 0.0271) | 0.760 |  |
| Age, years |  |  |  |  |  |
| 20-39 y | -0.0003 (-0.0367, 0.0361) | 0.987 | 0.0212 (-0.0219, 0.0642) | 0.338 |  |
| 40-59 y | -0.0155 (-0.0460, 0.0149) | 0.320 | 0.0084 (-0.0268, 0.0436) | 0.642 |  |
| 60 y | -0.0091 (-0.0344, 0.0162) | 0.483 | -0.0125 (-0.0392, 0.0141) | 0.359 |  |
| Race |  |  |  |  |  |
| Mexican American | **-0.0484 (-0.0952, -0.0015)** | **0.048** | -0.0133 (-0.0610, 0.0344) | 0.587 |  |
| Other Hispanic | 0.0205 (-0.0385, 0.0795) | 0.500 | -0.0453 (-0.1111, 0.0205) | 0.183 |  |
| Non-Hispanic White | -0.0067 (-0.0281, 0.0147) | 0.539 | 0.0066 (-0.0195, 0.0328) | 0.621 |  |
| Non-Hispanic Black | -0.0329 (-0.0753, 0.0096) | 0.133 | -0.0172 (-0.0617, 0.0273) | 0.450 |  |
| Other Race - including multi-racial | -0.0568 (-0.1454, 0.0317) | 0.214 | -0.0652 (-0.1376, 0.0073) | 0.083 |  |
| HLS, healthy lifestyle score. These variables were adjusted: age, sex, race, education, family income-to-poverty ratio, marital status, employment and insurance. | | | | |  |
|  |  |  |  |  |  |
|  |  |  |  |  |  |

| Table S2 Associations between lifestyle characteristics and immune-inflammation markers stratified by study year | | | | |  |
| --- | --- | --- | --- | --- | --- |
| Outcomes | HLS 2 vs. HLS 0-1 | | HLS 3-4 vs. HLS 0-1 | |  |
|  | Adjusted β (95%CI) | P value | Adjusted β (95%CI) | P value |  |
| **C-reactive protein (mg/dL)** |  |  |  |  |  |
| Year 1999-2006 | **-0.1692 (-0.2509, -0.0875)** | **<0.001** | **-0.2925 (-0.3925, -0.1925)** | **<0.001** |  |
| Year 2007-2014 | **-0.1310 (-0.2275, -0.0344)** | **0.010** | **-0.3340 (-0.4257, -0.2423)** | **<0.001** |  |
| **Platelet-lymphocyte ratio** |  |  |  |  |  |
| Year 1999-2006 | 0.0154 (-0.0190, 0.0498) | 0.387 | 0.0367 (-0.0014, 0.0748) | 0.066 |  |
| Year 2007-2014 | -0.0004 (-0.0224, 0.0216) | 0.969 | **0.0245 (0.0008, 0.0482)** | **0.049** |  |
| **Systemic immune-inflammation index** |  |  |  |  |  |
| Year 1999-2006 | -0.0329 (-0.0718, 0.0060) | 0.105 | -0.0338 (-0.0767, 0.0091) | 0.130 |  |
| Year 2007-2014 | **-0.0499 (-0.0749, -0.0249)** | **<0.001** | **-0.0923 (-0.1223, -0.0624)** | **<0.001** |  |
| **Neutrophil-lymphocyte ratio** |  |  |  |  |  |
| Year 1999-2006 | **-0.0371 (-0.071, -0.0032)** | **0.032** | -0.0246 (-0.0617, 0.0126) | 0.189 |  |
| Year 2007-2014 | **-0.0348 (-0.0552, -0.0144)** | **0.002** | **-0.0681 (-0.0919, -0.0444)** | **<0.001** |  |
| **Monocyte-lymphocyte ratio** |  |  |  |  |  |
| Year 1999-2006 | -0.0112 (-0.0386, 0.0162) | 0.428 | -0.0011 (-0.0345, 0.0324) | 0.951 |  |
| Year 2007-2014 | -0.0124 (-0.0358, 0.0110) | 0.304 | -0.0033 (-0.0299, 0.0232) | 0.807 |  |
| HLS, healthy lifestyle score. These variables were adjusted: age, sex, race, education, family income-to-poverty ratio, marital status, employment and insurance. | | | | |  |
|  |  |  |  |  |  |
|  |  |  |  |  |  |

| Table S3 Associations between lifestyle factors and immune-inflammation markers based on survey-weighted regression after excluding those without any PA, alcohol consumption or cigarette use. | | | | | | | | |  |
| --- | --- | --- | --- | --- | --- | --- | --- | --- | --- |
| Outcomes | Adjusted β (95%CI) | P value | Adjusted β (95%CI) | P value | Adjusted β (95%CI) | P value | Adjusted β (95%CI) | P value |  |
|  | HEI score (N=2229 for CRP; N=3588 for the other) | | Cigarettes per day (N=2229 for CRP; N=3588 for the other) | | Average drinks per day (N=2229 for CRP; N=3588 for the other) | | Physical activity** (N=2229 for CRP; N=3588 for the other) | |  |
| C-reactive protein (mg/dL) | **-0.0133 (-0.0180, -0.0086)** | **<0.001** | 0.0522 (-0.0246, 0.1290) | 0.188 | -0.0274 (-0.0567, 0.0018) | 0.071 | -0.0332 (-0.0869, 0.0205) | 0.230 |  |
| Platelet-lymphocyte ratio | -0.0002 (-0.0014, 0.0011) | 0.801 | 0.0042 (-0.0122, 0.0206) | 0.618 | **0.0090 (0.0015, 0.0165)** | **0.021** | -0.0061 (-0.0201, 0.0080) | 0.400 |  |
| Systemic immune-inflammation index | **-0.0032 (-0.0048, -0.0016)** | **<0.001** | 0.0227 (-0.0002, 0.0455) | 0.054 | -0.0028 (-0.0138, 0.0083) | 0.622 | -0.0126 (-0.0307, 0.0055) | 0.175 |  |
| Neutrophil-lymphocyte ratio | **-0.0016 (-0.0030, -0.0002)** | **0.029** | 0.0160 (-0.0022, 0.0342) | 0.087 | 0.0022 (-0.0070, 0.0113) | 0.643 | -0.0051 (-0.0191, 0.0090) | 0.481 |  |
| Monocyte-lymphocyte ratio | -0.00002 (-0.0012, 0.0012) | 0.974 | -0.0012 (-0.0168, 0.0145) | 0.886 | **0.0146 (0.0070, 0.0221)** | **<0.001** | -0.0082 (-0.0206, 0.0043) | 0.201 |  |
|  | HEI-high vs. HEI-low (N=2229 for CRP; N=3588 for the other) | | Ever-smoking vs. never-smoking (N=4119 for CRP; N=6798 for the other) | | Ever-drinking vs. never-drinking (N=3037 for CRP; N=4877 for the other) | | PA-high vs. PA-low (N=2229 for CRP; N=5118 for the other) | |  |
| C-reactive protein (mg/dL) | **-0.2911 (-0.4124, -0.1699)** | **<0.001** | **0.1527 (0.0667, 0.2387)** | **<0.001** | -0.1095 (-0.2222, 0.0033) | 0.062 | 0.0450 (-0.1539, 0.2439) | 0.659 |  |
| Platelet-lymphocyte ratio | -0.0002 (-0.0340, 0.0336) | 0.991 | **-0.0566 (-0.0825, -0.0306)** | **<0.001** | 0.0248 (-0.0108, 0.0603) | 0.175 | 0.0046 (-0.0223, 0.0315) | 0.737 |  |
| Systemic immune-inflammation index | **-0.0812 (-0.1296, -0.0329)** | **0.001** | 0.0022 (-0.0292, 0.0337) | 0.890 | -0.0240 (-0.0687, 0.0206) | 0.294 | -0.0328 (-0.0691, 0.0035) | 0.079 |  |
| Neutrophil-lymphocyte ratio | **-0.0418 (-0.0814, -0.0022)** | **0.041** | -0.0096 (-0.0348, 0.0156) | 0.458 | -0.0227 (-0.0621, 0.0168) | 0.262 | **-0.0525 (-0.0806, -0.0244)** | **<0.001** |  |
| Monocyte-lymphocyte ratio | -0.00009 (-0.0319, 0.0317) | 0.995 | -0.0196 (-0.0411, 0.0018) | 0.076 | 0.0217 (-0.0089, 0.0522) | 0.167 | **-0.0259 (-0.0482, -0.0035)** | **0.025** |  |
| **, metabolic equivalent times, calculated by multiplying time spent on each activity (minutes) and its metabolic equivalent score. These variables were adjusted: age, sex, race, education, family income-to-poverty ratio, marital status, employment and insurance. The other three lifestyle factors except for the analyzed one were also adjusted. All lifestyle factors were ln-transformed except for HEI. All outcomes were in-transformed. | | | | | | | | |  |
|  |  |  |  |  |  |  |  |  |  |
|  |  |  |  |  |  |  |  |  |  |

| Table S4 Associations between lifestyle factors and immune-inflammation markers based on survey-weighted regression in NHANES 2007-2014 (N=10202). | | | | | | | | | | | | |  |
| --- | --- | --- | --- | --- | --- | --- | --- | --- | --- | --- | --- | --- | --- |
| Outcomes | Adjusted β (95%CI) | P value | Adjusted β (95%CI) | P value | Adjusted β (95%CI) | P value | Adjusted β (95%CI) | P value | Adjusted β (95%CI) | P value | Adjusted β (95%CI) | P value |  |
|  | HEI score | | Cigarettes per day | | Average drinks per day | | Physical activity** | | Sleep hour | | Sedentary hour per day | |  |
| C-reactive protein (mg/dL) | **-0.0096 (-0.0130, -0.0061)** | **<0.001** | **0.0080 (0.0047, 0.0113)** | **<0.001** | **-0.0697 (-0.1078, -0.0316)** | **0.003** | **-0.0038 (-0.0055, -0.0021)** | **<0.001** | 0.0008 (-0.0254, 0.0270) | 0.952 | 0.0058 (-0.0004, 0.0119) | 0.086 |  |
| Platelet-lymphocyte ratio | **-0.0006 (-0.0013, -0.00000)** | **0.049** | -0.0005 (-0.0012, 0.0001) | 0.124 | 0.0068 (-0.0003, 0.0139) | 0.059 | 0.0002 (-0.0001, 0.0004) | 0.219 | 0.0019 (-0.0034, 0.0072) | 0.477 | **-0.0034 (-0.0058, -0.001)** | **0.005** |  |
| Systemic immune-inflammation index | **-0.0031 (-0.004, -0.0022)** | **<0.001** | **0.0018 (0.0009, 0.0027)** | **<0.001** | -0.0016 (-0.0113, 0.0081) | 0.751 | **-0.0004 (-0.0007, -0.00004)** | **0.031** | 0.0020 (-0.0053, 0.0092) | 0.596 | **0.0052 (0.0020, 0.0085)** | **0.002** |  |
| Neutrophil-lymphocyte ratio | **-0.0021 (-0.0028, -0.0014)** | **<0.001** | **0.0011 (0.0004, 0.0019)** | **0.004** | 0.0048 (-0.0034, 0.0130) | 0.249 | **-0.0003 (-0.0006, -0.00001)** | **0.042** | 0.0029 (-0.0032, 0.0090) | 0.350 | **0.0070 (0.0042, 0.0098)** | **<0.001** |  |
| Monocyte-lymphocyte ratio | -0.0001 (-0.0007, 0.0006) | 0.845 | -0.0004 (-0.0001, 0.0003) | 0.282 | **0.0208 (0.0139, 0.0276)** | **<0.001** | 0.00002 (-0.0002, 0.0003) | 0.849 | 0.0021 (-0.003, 0.0072) | 0.425 | **0.0048 (0.0025, 0.0071)** | **<0.001** |  |
|  | HEI-high vs. HEI-low | | Ever-smoking vs. never-smoking | | Ever-drinking vs. never-drinking | | LTPA-high vs. LTPA-low | | 6-8h vs. <6h or >8h | | Sedentary-high vs. Sedentary-low | |  |
| C-reactive protein (mg/dL) | **-0.2494 (-0.3448, -0.1540)** | **<0.001** | **0.1606 (0.0501, 0.2712)** | **0.013** | **-0.2381 (-0.3190, -0.1572)** | **<0.001** | **-0.3241 (-0.4316, -0.2166)** | **<0.001** | **-0.1102 (-0.1906, -0.0299)** | **0.016** | **0.1983 (0.1246, 0.2721)** | **<0.001** |  |
| Platelet-lymphocyte ratio | -0.0079 (-0.0243, 0.0085) | 0.347 | **-0.0489 (-0.0654, -0.0325)** | **<0.001** | 0.0013 (-0.0160, 0.0186) | 0.884 | -0.0026 (-0.0193, 0.0142) | 0.765 | 0.0127 (-0.0053, 0.0307) | 0.166 | -0.0114 (-0.0275, 0.0046) | 0.163 |  |
| Systemic immune-inflammation index | **-0.0594 (-0.0818, -0.0369)** | **<0.001** | **0.0299 (0.0074, 0.0524)** | **0.009** | -0.0204 (-0.0441, 0.0034) | 0.093 | **-0.0471 (-0.0699, -0.0242)** | **<0.001** | -0.0009 (-0.0255, 0.0237) | 0.943 | **0.0398 (0.0178, 0.0619)** | **<0.001** |  |
| Neutrophil-lymphocyte ratio | **-0.0441 (-0.0631, -0.0252)** | **<0.001** | **0.0201 (0.0011, 0.0391)** | **0.038** | -0.0153 (-0.0353, 0.0048) | 0.135 | **-0.0377 (-0.0570, -0.0183)** | **<0.001** | -0.0049 (-0.0257, 0.0159) | 0.647 | **0.0540 (0.0354, 0.0726)** | **<0.001** |  |
| Monocyte-lymphocyte ratio | -0.0014 (-0.0172, 0.0145) | 0.865 | **-0.0284 (-0.0443, -0.0125)** | **<0.001** | 0.0029 (-0.0139, 0.0197) | 0.735 | -0.0064 (-0.0225, 0.0098) | 0.440 | -0.0113 (-0.0287, 0.0062) | 0.205 | **0.0400 (0.0244, 0.0555)** | **<0.001** |  |
| **, metabolic equivalent times, calculated by multiplying time spent on each activity (hour) and its metabolic equivalent score. These variables were adjusted: age, sex, race, education, family income-to-poverty ratio, marital status, employment and insurance. The other five lifestyle factors except for the analyzed one were also adjusted. All lifestyle factors were ln-transformed except for HEI. All outcomes were in-transformed. | | | | | | | | | | | | |  |
|  |  |  |  |  |  |  |  |  |  |  |  |  |  |
|  |  |  |  |  |  |  |  |  |  |  |  |  |  |

| Table S5 Associations between lifestyle factors and immune-inflammation markers based on survey-weighted regression in NHANES 2007-2014 after excluding those without any LTPA, alcohol consumption or cigarette use. | | | | | | | | | | | | |  |
| --- | --- | --- | --- | --- | --- | --- | --- | --- | --- | --- | --- | --- | --- |
| Outcomes | Adjusted β (95%CI) | P value | Adjusted β (95%CI) | P value | Adjusted β (95%CI) | P value | Adjusted β (95%CI) | P value | Adjusted β (95%CI) | P value | Adjusted β (95%CI) | P value |  |
|  | HEI score (N=1038 for CRP; N=1961 for the other) | | Cigarettes per day (N=1038 for CRP; N=1961 for the other) | | Average drinks per day (N=1038 for CRP; N=1961 for the other) | | Physical activity** (N=1038 for CRP; N=1961 for the other) | | Sleep hour (N=1038 for CRP; N=1961 for the other) | | Sedentary time (N=1038 for CRP; N=1961 for the other) | |  |
| C-reactive protein (mg/dL) | **-0.0091 (-0.0173, -0.0009)** | **0.047** | 0.1230 (-0.0154, 0.2615) | 0.103 | -0.0214 (-0.0700, 0.0272) | 0.403 | -0.0423 (-0.1488, 0.0642) | 0.449 | -0.0089 (-0.0680, 0.0502) | 0.772 | **0.1650 (0.0609, 0.2690)** | **0.008** |  |
| Platelet-lymphocyte ratio | 0.0001 (-0.0017, 0.0019) | 0.927 | 0.0154 (-0.0086, 0.0395) | 0.215 | **0.0125 (0.0035, 0.0215)** | **0.009** | -0.0013 (-0.0179, 0.0153) | 0.877 | 0.0093 (-0.0045, 0.0230) | 0.192 | **-0.0299 (-0.0582, -0.0015)** | **0.045** |  |
| Systemic immune-inflammation index | **-0.0039 (-0.0063, -0.0014)** | **0.004** | 0.0311 (-0.0044, 0.0665) | 0.093 | 0.0064 (-0.0089, 0.0216) | 0.419 | -0.0055 (-0.0289, 0.0179) | 0.647 | -0.0011 (-0.0215, 0.0193) | 0.917 | -0.0006 (-0.0379, 0.0367) | 0.977 |  |
| Neutrophil-lymphocyte ratio | **-0.0026 (-0.0047, -0.0005)** | **0.022** | 0.0117 (-0.0165, 0.0398) | 0.420 | 0.0125 (-0.0011, 0.0261) | 0.079 | 0.0060 (-0.0152, 0.0271) | 0.583 | 0.0019 (-0.0141, 0.0178) | 0.822 | 0.0049 (-0.0281, 0.0379) | 0.771 |  |
| Monocyte-lymphocyte ratio | -0.0006 (-0.0023, 0.0011) | 0.501 | 0.00001 (-0.022, 0.0221) | 0.999 | **0.0221 (0.0130, 0.0312)** | **<0.001** | -0.0014 (-0.0173, 0.0145) | 0.866 | 0.0038 (-0.0094, 0.0169) | 0.579 | -0.0129 (-0.0418, 0.0161) | 0.388 |  |
|  | HEI-high vs. HEI-low (N=1038 for CRP; N=1961 for the other) | | Ever-smoking vs. never-smoking (N=1964 for CRP; N=3857 for the other) | | Ever-drinking vs. never-drinking (N=1409 for CRP; N=2658 for the other) | | LTPA-high vs. LTPA-low (N=1038 for CRP; N=3486 for the other) | | 6-8h vs. <6h or >8h (N=1038 for CRP; N=1961 for the other) | | Sedentary-high vs. Sedentary-low (N=1038 for CRP; N=1962 for the other) | |  |
| C-reactive protein (mg/dL) | **-0.2366 (-0.4315, -0.0417)** | **0.032** | 0.1390 (-0.0080, 0.2859) | 0.085 | -0.1879 (-0.3786, 0.0028) | 0.069 | 0.0363 (-0.2409, 0.3134) | 0.802 | -0.0479 (-0.2055, 0.1098) | 0.561 | **0.1809 (0.0301, 0.3317)** | **0.031** |  |
| Platelet-lymphocyte ratio | 0.0041 (-0.0428, 0.0510) | 0.864 | **-0.0614 (-0.0964, -0.0264)** | **0.001** | -0.0067 (-0.0551, 0.0416) | 0.787 | -0.0205 (-0.0520, 0.0110) | 0.208 | 0.0091 (-0.0349, 0.0531) | 0.683 | **-0.0451 (-0.0806, -0.0096)** | **0.017** |  |
| Systemic immune-inflammation index | **-0.0816 (-0.1506, -0.0125)** | **0.025** | 0.0088 (-0.0299, 0.0475) | 0.659 | **-0.0843 (-0.1485, -0.0202)** | **0.012** | **-0.0585 (-0.1002, -0.0168)** | **0.008** | 0.0033 (-0.0621, 0.0686) | 0.923 | -0.0112 (-0.0622, 0.0399) | 0.671 |  |
| Neutrophil-lymphocyte ratio | -0.053 (-0.1102, 0.0042) | 0.076 | -0.0015 (-0.0325, 0.0295) | 0.926 | **-0.0815 (-0.1382, -0.0248)** | **0.007** | **-0.0559 (-0.0876, -0.0241)** | **0.001** | 0.0048 (-0.0509, 0.0605) | 0.868 | 0.0081 (-0.0412, 0.0575) | 0.748 |  |
| Monocyte-lymphocyte ratio | -0.0124 (-0.0562, 0.0314) | 0.581 | -0.0200 (-0.0486, 0.0086) | 0.178 | -0.0107 (-0.0522, 0.0309) | 0.618 | **-0.0356 (-0.0618, -0.0094)** | **0.011** | -0.0270 (-0.0664, 0.0124) | 0.186 | -0.0032 (-0.0452, 0.0387) | 0.881 |  |
| **, metabolic equivalent times, calculated by multiplying time spent on each activity (minutes) and its metabolic equivalent score. These variables were adjusted: age, sex, race, education, family income-to-poverty ratio, marital status, employment and insurance. The other five lifestyle factors except for the analyzed one were also adjusted. All lifestyle factors were ln-transformed except for HEI. All outcomes were in-transformed. | | | | | | | | | | | | |  |
|  |  |  |  |  |  |  |  |  |  |  |  |  |  |
|  |  |  |  |  |  |  |  |  |  |  |  |  |  |
